# TRIM65 Promotes Cervical Cancer Through Selectively Degrading p53-Mediated Inhibition of Autophagy and Apoptosis

**DOI:** 10.3389/fonc.2022.853935

**Published:** 2022-03-24

**Authors:** Xiao-Yu Wang, Hai-Wei Mao, Xiao-Hui Guan, Qi-Ming Huang, Zhen-Ping Yu, Jie Wu, Hui-Lan Tan, Feng Zhang, Xuan Huang, Ke-Yu Deng, Hong-Bo Xin

**Affiliations:** ^1^ The National Engineering Research Center for Bioengineering Drugs and the Technologies, Institute of Translational Medicine, Nanchang University, Nanchang, China; ^2^ College of Life Science, Nanchang University, Nanchang, China; ^3^ Institute of Geriatrics, Jiangxi Provincial People’s Hospital, Nanchang, China; ^4^ Outpatient Department, The First Affiliated Hospital of Nanchang University, Nanchang, China

**Keywords:** TRIM65, ubiquitination, autophagy, apoptosis, cervical cancer

## Abstract

Tripartite motif containing 65 (TRIM65) is an E3 ubiquitin ligase that has been implicated in a variety of cellular processes as well as tumor progression, but its biological role and the underlying mechanism in cervical cancer is unclear. Here, we reported that TRIM65 expression in human cervical cancer tissues was significantly higher than that in the adjacent normal cervical tissues, and TRIM65 knockdown enhanced autophagic flux and cell apoptosis, but not cell cycle, to dramatically inhibit the proliferation and migration of cervical cancer cells. Furthermore, our experiments showed that TRIM65 exhibited oncogenic activities *via* directly targeting p53, a tumor suppressor and a common upsteam regulator between autophagy and apoptosis, promoting ubiquitination and proteasomal degradation of p53. Taken together, our studies demonstrated that TRIM65 knockdown promotes cervical cancer cell death through enhancing autophagy and apoptosis, suggesting that TRIM65 may be a potential therapeutic target for cervical cancer clinically.

## Introduction

Cervical cancer, the most frequent human papillomavirus (HPV)-associated cancer, is the fourth most common cancer in women globally and the second cause of cancer-related death among women in developing countries (1, 2). The prognosis of this disease is poor due to a high metastasis or recurrence. Although the most common therapies including radiotherapy, chemotherapy, immunotherapy and targeted therapy have been able to slightly extend overall survival, these methods remain insufficient, expensive and cause severe side effects (3, 4). Squamous cell carcinoma and adenocarcinoma, two main histological types of cervical cancers, had 80% to 90% and 10% to 20% of the diagnosed cervical cancers, respectively (5, 6).

Studies demonstrated that HPV infection is not only an important risk factor for the invasive squamous cell carcinomas, but also for intraepithelial lesions, and HPV DNA has been found in more than 90% of preinvasive and invasive lesions, implicating the significance of HPV in the pathogenesis of cervical cancer (7). Autophagy is one of the major intracellular degradation systems in addition to the ubiquitin-proteasome system, in which autophagy utilizes the lysosome as a garbage machine for digestion, whereas the ubiquitin mediates the protein degradation by proteasomes (8). Studies indicated that autophagy has a critical inhibitory effect on HPV infection in tumorigenesis and development of cervical cancer (9). However, HPV also suppressed autophagy upon infection to avoid early clearance of viral particles, resulting in aggravation of infectivity by accelerating the proliferation and transformation of the infected cells (10). In early stage of cervical cancer, the expression levels of essential autophagic proteins, such as ATG3 and BECN1, are significantly lower than that in adjacent normal tissues, indicating that autophagy plays a role in the tumor inhibition.

Tripartite motif (TRIM) proteins, which are defined by containing a RING domain, one or two B-boxes domain and a coiled-coil domain in the amino-terminal region (11), have various functions in cellular processes including intracellular signaling, development, apoptosis, protein quality control, innate immunity, autophagy and carcinogenesis (12, 13). The TRIM family proteins are conserved throughout the metazoan kingdom, in which more than 80 family members have been identified in human, and most of them possess E3 ubiquitin ligase activities due to their RING-finger domain (13). An important role of TRIM proteins is to mediate both the engulfment and degradation of pathogens within autophagosomes in innate immune response (14). It is noteworthy that as receptors and regulators of autophagy, TRIM proteins have been directly involved in multiple steps of the autophagic process including upstream signaling pathways, regulation of autophagy-related genes expression and formation of autophagosome (15). Moreover, TRIM proteins also play an important role in transporting pathogens to autophagosomes for degradation in the context of tumorigenesis.

Human TRIM65, a member of TRIM family proteins, is a 517 amino acids length protein containing an N-terminal RING domain, a B-box, and a coiled-coil domain and a SPRY domain. Studies showed that TRIM65 acts as an ubiquitin E3 ligase, targeting p53 (16), ANXA2 (17), Axin1 (18), ARHGAP35 (19), TNRC6 (20), MDA5 (21) and VCAM-1 (22) to regulate carcinogenesis, innate immunity, miRNA pathway and inflammation. Since HPV status of cervical cancer is extensively connected with the immune landscape (23), we intend to explore the role and mechanism of TRIM65 in HPV-associated cervical cancer.

In the present study, we observed that TRIM65 expression was up-regulated in cervical tissues, and knockdown of TRIM65 inhibited the proliferation and migration of the cervical cancer cells by promoting autophagy and autophagy-related apoptosis and the inhibitory effects were almost completely inhibited by autophagy inhibitor, suggesting that TRIM65-downregualted autophagic flux may be closely associated with cervical carcinogenesis. In view of the involvement of tumor suppressor, p53, in autophagy convergence and apoptosis, we further demonstrated that TRIM65 promoted p53 ubiquitination and degradation, suggesting that TRIM65-mediated p53 degradation played a critical role in carcinogenesis of cervical cancer cell. These data indicated that TRIM65 knockdown-induced autophagy inhibited cervical cancer by delivering the autophagosomes for degradation in the cancer cells, suggesting that TRIM65 may be a potential target for treatment of cervical cancer.

## Materials and Methods

### Tissue Samples

The paraffin-embedded tissues of 16 cervical squamous cell carcinomas, 8 cervical adenocarcinomas, and 5 adjacent non-tumorous cervical tissues were obtained from the Department of Pathology, the First Affiliated Hospital of Nanchang University. The age range of patients was 40-65. This study was approved by the Ethics Committee of the First Affiliated Hospital of Nanchang University. All the experiments were conducted in accordance with the principles of the Helsinki Declaration.

### Survival Analysis of TRIM65

The GEPIA (http://gepia.cancer-pku.cn/index.html) website was used to preform survival analysis. The patients with cervical cancer were divided into a high expression group and a low expression group based on the median expression level of TRIM65. The Kaplan-Meier method was applied to plot survival curves. The survival plot showed that the patients with low TRIM65 expression were associated with longer survival time.

### Cell Culture and Reagents

HeLa and SiHa cells were purchased from Shanghai Cell Biology Institute of Chinese Academy of Sciences (Shanghai, China) and cultured in Dulbecco’s Modified Eagle Medium (DMEM) supplemented with 10% fetal bovine serum (FBS, Thermo Fisher Scientific, USA) and 100 U/ml penicillin and streptomycin. All the cells were cultured at 37°C in a humidified atmosphere of 5% CO_2_. For immunofluorescence assay, HeLa cells expressing GFP-LC3 were transiently transfected with TRIM65 siRNAs. After 42 h, the cells were further treated with or without CQ for 6 hours and then analyzed by fluorescence microscopy. The cells with more than ten GFP-LC3 puncta were regarded as positive cells, and the percentage of the cells with LC3 puncta per field was calculated according to a previously published protocol (24). MG132 (Sigma, USA), Cycloheximide (CHX) (Sigma, USA), Chloroquine (CQ) (Sigma, USA) and Lipofectamine 3000 (Thermo Fisher Scientific, USA) were bought from the suppliers. The sequences of the siRNAs targeting human TRIM65 mRNA in this study were as follows: siRNA-1: 5′-TGGCCTCAGGCTGCCTCACCTTCTA-3′; siRNA-2: 5′-GGAAACTCTGGCAGAATTATCGCAA-3′; siRNA-3: 5′-TCAGCGCCAACCGTCACTTCTATCT-3′.

### Colony Formation and Transwell Assays

For colony formation assays, cells were collected and seeded into six-well plates at a density of 1000 cells/well and then incubated at 37°C for 6 days. Colonies were fixed with 4% paraformaldehyde and stained with 0.1% crystal violet. For transwell assays, cells were resuspended in the upper compartment of transwell system (Coring, USA) in serum-free medium, while the lower chambers were filled 15% FBS as a chemoattractant. After 24 h of incubation, the cells on the bottom of the chamber were fixed, stained, and counted. For the experiments of TRIM65 knockdown and overexpression, the initial cells were 5 χ 10^5^ cells/well and 10^4^ cells/well, respectively.

### Western Blot Analysis

Cells were harvested and lysed in RIPA buffer containing protease inhibitor cocktail for 30 min at 4°C and total cell extracts were subjected to 10% or 12% SDS-PAGE, then transferred to PVDF membranes. The membranes were blocked with 5% skim milk and incubated with primary antibodies anti-β-actin (CST, 3700T), anti-LC3B (CST, 2775S), anti-p62 (CST, 39749S), anti-AMPK(CST, 5831), anti-ATG5(CST, 12994), p-mTOR (CST, 5536), mTOR (CST, 2983), p-S6 (CST, 9205), anti-Bax (Santa Cruz, sc-20067), anti-Bcl_2_ (Santa Cruz, sc-7382), S6 (Santa Cruz, sc-8418), anti-p53 (OriGene, UM570053), anti-TRIM65 (Abcam, HPA021578), anti-GAPDH (Abcam, ab8245) at 4°C overnight, followed by incubation with horseradish peroxidase–linked anti-mouse or anti-rabbit secondary antibodies for 1 h at room temperature. Images were quantified using a digital gel image analysis system TANON 5500 (Shanghai, China) and the protein intensities were quantified by Tanon GIS software.

### Cell Cycle and Apoptosis Analysis by Flow Cytometry

For cell cycle assays, cells were transfected with siRNA for 48 h and then digested and washed with ice-cold PBS. The cells were fixed in 70% ethanol for 30 min on ice, then washed twice in PBS and incubated with propidium iodide (PI) (Dojindo, Japan) for 30 min. Flow cytometry (BD Biosciences, USA) was implemented to assess Intensities of fluorescence signals. Data were analyzed by with BD Jazz.

For the apoptosis assays, cells were transfected with siRNA for 48 h, and then, collected and washed with ice-cold PBS. Staining was performed using the Annexin V-FITC Apoptosis Detection Kit (Dojindo, Japan), according to the manufacturer’s instructions. After incubation for 15 min at room temperature, the cells were run and measured on a flow cytometer (BD Biosciences, USA).

### Immunohistochemistry

Tissue specimens from patients with human cervical cancer were stained for the expression of TRIM65 (Abcam, USA) according to a previously published protocol. Expression levels were analyzed by a proportion of immunopositive staining area, and assessed by a technician blinded to the identity of the tissue cores.

### Co-Immunoprecipitation

Briefly, the cells were collected and lysed on ice for 30 min with lysis buffer with protease inhibitors and phosphatase inhibitors. The cell lysates were incubated with gentle rocking for overnight at 4°C with anti-FLAG antibody or IgG crosslinked to protein A/G beads (Roche, Germany). Then, the immune-blotting precipitates were washed and subjected to western blotting using p53 antibodies.

### Statistical Analysis

Statistical analysis was performed with GraphPad software. The results were analyzed by paired t-test, or analysis of variance. Data from three separate experiments are presented as mean ± SD. P-value<0.05 was considered to be statistically significant.

## Results

### TRIM65 Expression Is Upregulated in Human Cervical Cancer Tissues

**Figure 1:**
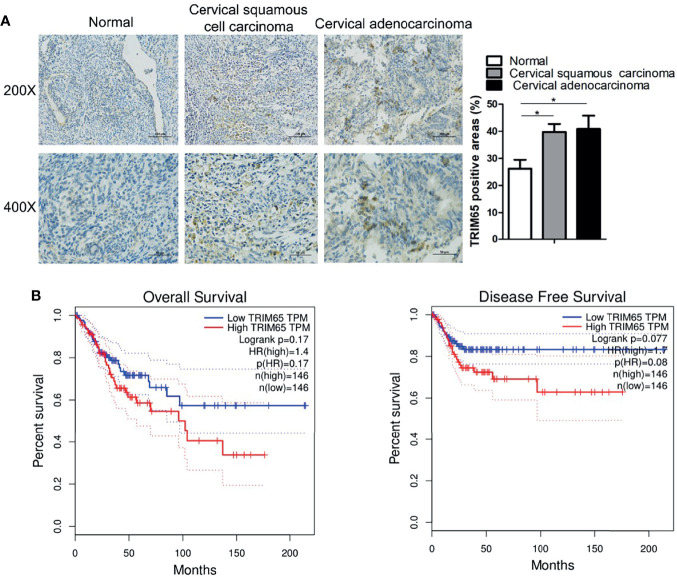
TRIM65 expression is increased in human cervical cancer tissues. **(A)** Representative images of TRIM65 by IHC staining were shown on human cervical tumor and non-tumor tissues from the First Affiliated Hospital of Nanchang University. Scale bar = 100 μm (Upper panel), scale bar = 50 μm (Bottom panel). **(B)** The prognostic value of TRIM65 protein for overall survival (left) and disease-free survival (right) was determined by Kaplan–Meier analyses. Data is from TCGA’s cervical carcinoma data set. Data are presented as mean ± SD. The experiments were repeated three times independently and the data of one representative experiment was shown. *p < 0.05.

To investigate the role of TRIM65 in cervical cancer, the expressions of TRIM65 in human cervical cancer tissues were examined and the survival analysis was performed by using the GEPIA website (http://gepia.cancer-pku.cn/index.html). As shown in **Figure 1A**, TRIM65 expression was significantly higher in cervical cancer tissues than that in adjacent normal cervical tissues from the First Affiliated Hospital of Nanchang University. For survival analysis, the patients with cervical cancer from TCGA’s cervical carcinoma data set were divided into a high expression group and a low expression group based on median expression level of TRIM65 and the Kaplan-Meier method was applied to plot overall and disease-free survival curves. The results showed that the patients with low TRIM65 expression were tendentiously associated with longer survival time or disease-free survival time although there were no significant statistical differences (**Figure 1B**), suggesting that TRIM65 may be involved in carcinogenesis of human cervical cancer.

### TRIM65 Promotes the Growth and Migration of Cervical Cancer Cells

**Figure 2:**
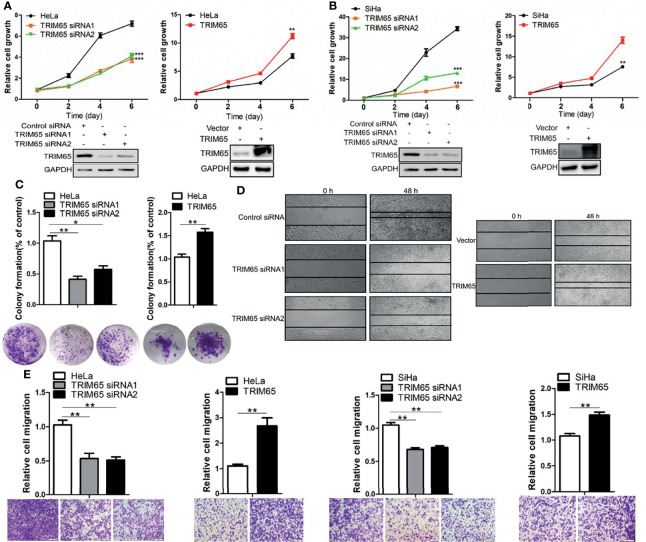
TRIM65 facilitates the proliferation and migration of cervical cancer cells. HeLa **(A)** and SiHa **(B)** cells were transfected with siRNAs or TRIM65 overexpression vector. After 24 h, the cells were seeded in 96-well plates at 2000 cells per well. At the indicated times, related cell growth was measured by CCK-8 (cell counting kit-8) in HeLa and SiHa cells with TRIM65 depletion (left) or overexpression (right). The statistical data was shown (Top panel). Immunoblot experiment was used to check the knockdown and overexpression efficiency of TRIM65 in both cell lines (Bottom panel). **(C)** HeLa cells were transfected with TRIM65 overexpression vector or siRNAs to determine cell proliferation by colony formation assay. A quantitative analysis of the colony was shown in the bar diagram. **(D)** Wound healing assay was used to confirm the effects of TRIM65 knockdown or overexpression on cell migration. **(E)** HeLa and SiHa cells were transfected with TRIM65 overexpression vector or siRNAs to show that TRIM65 was capable of enhancing cell migration by transwell assays. Bar diagram represents relative capable of cell migration. Scale bar = 200 μm. Data are presented as mean ± SD. The experiments were repeated three times independently and the data of one representative experiment was shown. *p < 0.05; **p < 0.01, ***p < 0.001.

To investigate whether TRIM65 is involved in the malignant progression of cervical cancer, TRIM65 was knockdown using siRNA in squamous cell carcinoma (SiHa) and adenocarcinoma (HeLa), respectively. As shown in **Figures 2A, B**, TRIM65 knockdown significantly inhibited cell proliferation in two cell lines. Conversely, TRIM65 overexpression remarkably enhanced cell growth compared with the control groups. The effect of TRIM65 on cell proliferation was further examined by colony formation assays, in which TRIM65 overexpression significantly increased the colony formation, whereas knockdown of TRIM65 remarkably decreased the formation of colonies (**Figure 2C**). Next, the wound healing assay was performed to assess the impact of TRIM65 on cell migration. TRIM65 knockdown markedly inhibited cell migration and in contrast, overexpression of TRIM65 promoted the increase of cell migration (**Figure 2D**). Similarly, the transwell assay also showed that knockdown of TRIM65 in HeLa and SiHa cells significantly reduced the movement of the cells from upper chamber into the lower chamber. Conversely, overexpression of TRIM65 in the cells remarkably enhanced the migration of the cells compared with the corresponding controls (**Figure 2E**), suggesting that TRIM65 was able to promote the migration of tumor cells. All these results indicated that TRIM65 functioned as an oncogene that promoted the growth and migration of the cervical cancer cells *in vitro*.

### Knockdown of TRIM65 Inhibits the Proliferation of Cervical Cancer Cells by Enhancing Autophagic Flux

**Figure 3:**
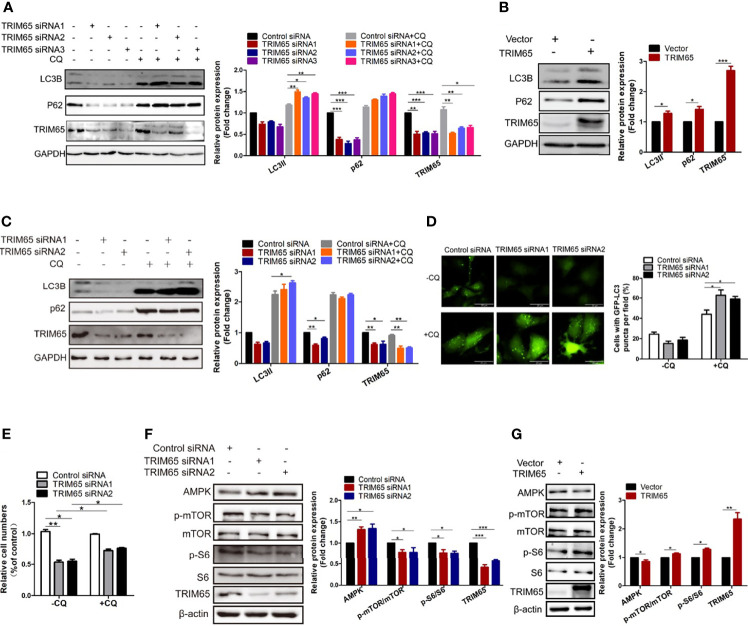
Knocking down TRIM65 induces autophagy in cervical cancer. HeLa **(A)** and SiHa **(C)** cells with TRIM65 depletion were harvested and lysed for western blot analysis using LC3- and p62-specific antibodies in the presence or absence of 20μM CQ for 6 h. GAPDH was used as a loading control. Relative protein expression over GAPDH was quantified, and the statistical data was shown (Right panel). **(B)** HeLa cells were transfected with TRIM65 overexpression vector to determine the expression of LC3 and p62 by western blot analysis. Relative protein expression over GAPDH was quantified, and the statistical data was shown (Right panel)**. (D)** HeLa cells expressing GFP-LC3B were transiently transfected with TRIM65 siRNAs. 42 h later, the cells were treated with or without CQ for 6 hours and then analyzed by fluorescence microscopy. GFP-LC3B puncta were counted under 40× magnification. Scale bar = 20 μm. **(E)** HeLa cells were transfected with control siRNA or TRIM65 siRNA1, TRIM65 siRNA2. 24 h later, cells were seeded in 24-well plates in 0.5 mL complete culture medium treated with or without CQ. On the 4th day, cells were trypsinized and counted. The statistical data was shown. The autophagy related proteins were analyzed by western blot using indicated antibodies in cells with TRIM65 knockdown **(F)** and overexpression **(G)**. Relative protein expression over β-actin was quantified, and the statistical data was shown (Right panel). Data are presented as mean ± SD. The experiments were repeated three times independently and the data of one representative experiment was shown. *p < 0.05; **p < 0.01, ***p < 0.001.

Since HPV is the most important factor for pathogenesis of cervical cancer and autophagy plays a key role in defending the infection of the virus in cervical carcinogenesis, we examined whether TRIM65 regulated autophagic flux. As shown in **Figure 3A**, knockdown of TRIM65 in HeLa cells significantly inhibited the protein expression of LC3 and P62, the canonical markers for autophagy. It is known that autophagy is a highly dynamic process. The protein levels of LC3 might not be enough to confirm the change of autophagic flux, since the expressions of LC3-II may be affected by reduced autophagosomes or the consumption of this protein *via* an intense autophagy flux. In order to assess autophagic flux, we applied the autophagy inhibitor-chloroquine (CQ) to prevent lysosomal degradation. The expression levels of LC3-II in TRIM65 silenced cells were significantly increased when compared with control siRNA group in the presence of CQ, indicating TRIM65 knockdown enhances autophagic flux. Conversely, TRIM65 overexpression dramatically elevated the expressions of LC3 and P62 in the cells compared with control groups (**Figure 3B**). The similar results were also obtained in SiHa cells (**Figure 3C**). These results suggested that the inhibitory effect of TRIM65 on autophagy may be not directly associated with degradations of LC3 and P62/SQSTM1. To further validate our results, HeLa cells expressing GFP-LC3 were constructed to detect the formation of autophagosomes by observing punctate structures. As shown in **Figure 3D**, LC3B puncta were decreased in TRIM65 silenced cells, while the puncta were dramatically increased when treated with CQ, which was consistent with the results from Western blot. Furthermore, we investigated whether TRIM65 knockdown-mediated the inhibition of cervical cancer cell proliferation was the consequence of increased autophagic flux. The results showed that TRIM65 knockdown markedly inhibited cell proliferation compared with the control group, in which the suppression was significantly reversed by CQ (**Figure 3E**). Furthermore, the Western blots results further confirmed that the protein level of AMPK was increased in TRIM65 silenced cells along with a decrease in p-mTOR and p-S6 (**Figure 3F**), whereas TRIM65 overexpression significantly promoted the phosphorylation of both mTOR and S6 (**Figure 3G**). These results demonstrated that the inhibitory effect of TRIM65 knockdown on cervical cancer cell proliferation might be related to inhibiting mTOR pathway, and in turn, to activate autophagy initiation, a classic signaling pathway.

### Knockdown of TRIM65 Enhances Autophagy-Related Apoptosis of Cervical Cancer Cells

In general, suppression of cancer is related to several mechanisms including induction of autophagy, apoptosis and cell cycle arrest. Autophagy is an important and evolutionarily conserved mechanism for maintaining cellular homeostasis, which has been shown to play a key role in TRIM65-induced cervical cancer cell proliferation from our data. However, it is still unclear whether the inhibitory effect of TRIM65 knockdown on cervical cancer is associated with apoptosis and cell cycle.

**Figure 4:**
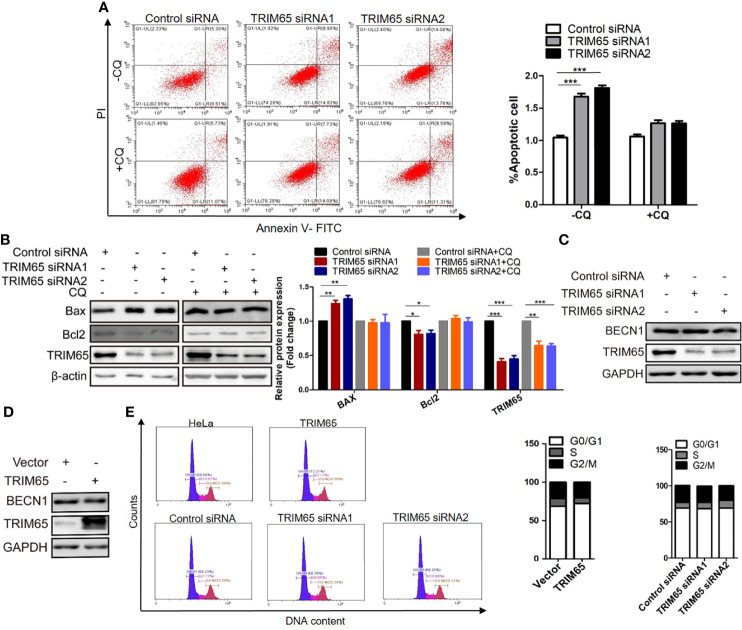
The effect of TRIM 65 on the cell apoptosis and cycle. **(A)** Cells were transiently transfected with siRNA-NC or siRNA-TRIM65. 42 h later, the cells were treated with or without 20 μM CQ for 6 h, then the cell apoptosis was analyzed by Annexin V/PI staining. Bar diagram represents percentage of apoptotic cells. NC, negative control. **(B)** Bax and Bcl_2_ were detected in the presence or absence of CQ by Western blotting. Relative protein expression over β-actin was quantified, and the statistical data was shown (Right panel). The expression of BECN1 was analyzed by western blot in HeLa cells with TRIM65 knockdown **(C)** or overexpression **(D)**. **(E)** Cell cycle profile was analyzed using flow cytometry in cells with TRIM65 knockdown and overexpression. Data are presented as mean ± SD. The experiments were repeated three times independently and the data of one representative experiment was shown. *p < 0.05; **p < 0.01, ***p < 0.001.

Thus, the effects of TRIM65 on cell apoptosis were examined in HeLa cells using TRIM65 siRNAs. Our results from flow cytometric analysis showed that downregulation of TRIM65 significantly increased cell apoptotic rates in comparison with siRNA-NC (**Figure 4A**). In addition, the increased expression of Bax and decreased expression of Bcl2, two core proteins involved in the apoptosis, were observed by Western blot in cells with TRIM65 knockdown (**Figure 4B**). Recently, studies reported that induction of apoptosis was a common event in cancer cells in response to autophagy activation (25, 26). Therefore, to clarify if there was an intrinsic link between autophagy and apoptosis induced by TRIM65 knockdown, we examined the apoptotic effect of TRIM65 siRNA on cervical cancer cells treated with autophagy inhibitor CQ. We observed that TRIM65 siRNA remarkably promoted cell apoptosis which was significantly reversed by CQ treatment (**Figures 4A, B**). These results indicated that autophagy was involved in TRIM65 knockdown-mediated cell apoptosis. Interestingly, previous studies demonstrated that autophagy was dependent on BECN1 in HeLa cells (27), but our results showed that the expressions of BECN1 were not affected by TRIM65 knockdown or overexpression (**Figures 4C, D**). Interestingly, it has been reported that Bcl2, the anti-apoptotic protein, disrupted the BECN1/Vps34 complexes through physically interacting with BECN1, a conserved autophagy protein containing a Bcl2 homology (BH) 3 region, and then inhibited autophagy (28), in which BECN1/Vps34 complexes were required for early stages of autophagosomal formation. TRIM65 siRNA-induced reduction of Bcl2 triggered autophagy by disrupting the Bcl-2/BECN1 complex. Therefore, we speculate that TRIM65 knockdown may induce autophagic flux by alleviating the suppression of Bcl2 on BECN1.

In addition, we further investigate the effects of TRIM65 knockdown or overexpression on cell cycle. As shown in **Figure 4E**, TRIM65 has no significant effect on S phase and the ratios of G0/G1 and G2/M phase cells. Collectively, these data suggested that the TRIM65 knockdown significantly activated autophagy and promoted apoptosis, resulting in the increase of cervical cancer cell death.

### TRIM65 Inhibits Autophagy *via* Selectively Degrading p53

**Figure 5:**
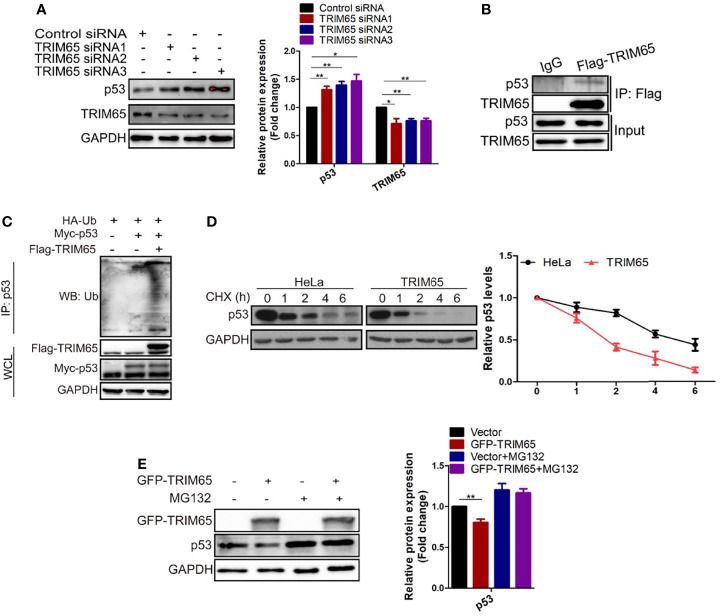
TRIM65 promotes ubiquitination and degradation of p53. **(A)** Western blot analysis of the expression levels of p53 in HeLa cells with TRIM65 knockdown. GAPDH was used as a loading control. Relative protein expression over GAPDH was quantified, and the statistical data was shown (Right panel). **(B)** IP and western blot analysis of the interaction of Flag-TRIM65 with p53 in the lysates of HeLa. **(C)** Cells were co-transfected with p53 plus HA-ubiquitin and/or Flag-TRIM65 as indicated. Then the cell lysates were immunoprecipitated with p53 antibody, the ubiquitination was detected using anti-ubiquitin antibody. **(D)** HeLa cells were transfected with vector plasmid or TRIM65 and treated with 25 μg/ml CHX for different time points, followed by western blot analysis (Top panel). The related protein levels at the indicated treatment times (Bottom panel). **(E)** HeLa cells with TRIM65 overexpression were treated with or without MG132, and then immunoblotted with antibodies against p53. Relative protein expression over GAPDH was quantified, and the statistical data was shown (Right panel). Data are presented as mean ± SD. The experiments were repeated three times independently and the data of one representative experiment was shown. *p < 0.05; **p < 0.01.

The results from our studies above and others indicated that autophagy and apoptosis usually occur in the same cell, mostly in the order that autophagy precedes apoptosis (29, 30). Furthermore, several proteins such as p53, are not only present in autophagy pathways but in apoptosis signal transduction (31–33). As mentioned above, TRIM65 knockdown exhibits anti-proliferative activity by both activating autophagy and apoptosis in cervical cancer. Accordingly, we examined the effects of TRIM65 on p53 tumor suppressor, a common upsteam protein between autophagy and apoptosis. The results from Western blot analysis showed that knockdown of TRIM65 significantly up-regulated the expression of p53 (**Figure 5A**). In addition, p53 was pulled down by Flag antibody of TRIM65, but not by IgG, in a co-immunoprecipitation experiment (**Figure 5B**), suggesting that there might be an interaction between p53 and TRIM65. Since TRIM65 is an E3 ligase, next, the Ub and p53 plasmid were co-transfected into HeLa cells with or without TRIM65 to determine the ubiquitination level of lysates. The results showed that TRIM65 overexpression dramatically increased the amount of poly-ubiquitinated p53 (**Figure 5C**). Furthermore, the p53 protein stability was determined in the condition of TRIM65-mediated ubiquitination. As presented in **Figure 5D**, the half-life of p53 protein was greatly shortened by TRIM65 overexpression. Importantly, MG132, a proteasome inhibitor, rescued TRIM65-mediated downregulation of p53, suggesting that p53 might be subjected to the ubiquitin–proteasome dependent degradation (**Figure 5E**). These findings indicated the TRIM65 played a vital role in the carcinogenesis of cervical cancer by simultaneously inhibiting the autophagic flux and apoptosis *via* ubiquitylation and degradation of p53, suggesting TRIM65 expression level may be useful for survival prediction of cervical cancer patients.

## Discussion

In the present study, we demonstrated for the first time that TRIM65 was closely related to carcinogenesis of cervical cancer. Multiple factors including oncogene or tumor suppressors, metabolism, autophagic flux, epigenetic mechanism and promoter methylation occur frequently in cancer progression (34–36). Particularly, autophagy manipulation is an important co-factor of several HPV-derived malignancies including cervical cancer (10, 37). Studies showed that inhibition of autophagy promoted HPV lifecycle and carcinogenesis, and that therapeutic strategies aimed at restoring the autophagic response could be valuable for suppressing HPV-mediated cervical cancer (10). Most members of TRIM protein family serve as E3 ligases which plays an important role in the physiological and pathological conditions. Interestingly, TRIM proteins (such as TRIM 5α, 6, 16, 17, 20, 22, 49, and 55) broadly affect autophagy by interacting with autophagy regulators and effectors including ULK1, beclin 1, mAtg8s and p62 (15, 38, 39). However, the effect and underlying mechanism of TRIM65 E3 ligase on cervical cancer carcinogenesis remains unclear.

We provided strong evidence that TRIM65 knockdown remarkably restrained the proliferation and migration of cervical cancer cell, while the pro-tumor activity was observed in the cells with TRIM65 overexpression. Further experiments indicated that TRIM65 knockdown increased autophagic flux by accelerating degradation of autolysosome which is essential for inhibition of cervical cancer. Consistent with this, previous studies showed that autophagy as a tumor suppressor inhibited HPV infection at the early stages of cervical carcinogenesis *via* maintaining genomic stability and integrity (40, 41). In addition, we observed that TRIM65 knockdown promoted autophagy-related apoptosis of cervical cancer cells, but did not affect the cell cycle. Thus, the interactions and molecular mechanisms between autophagy and apoptosis are worth to be further explored in cervical cancer cells with TRIM65 depletion. Autophagy, a homeostatic process whereby cytoplasmic cargo including damaged organelles and toxic aggregates was delivered to lysosome for degradation, play an extensive role in health and disease states (42). Apoptosis, known as programmed cell death, removed superfluous, aged, damaged or ectopic cells (43). Both autophagy and apoptosis are the major models of cell death that play important roles in tissue homeostasis and various diseases (44, 45). A complex cross-talk between two biological processes is often triggered by similar stimuli, such as growth factor, metabolic stress and signaling pathways. Accumulating evidence reveals that autophagy often occurs prior to apoptosis in the same cell. In general, autophagy could block the induction of apoptosis, but in special cases, autophagy could help to stimulate apoptosis by upregulating caspase (46). Autophagy and apoptosis can cooperate, antagonize or assist each other, and function as a switch affecting cell fate (47). In our studies, the decrease of Bcl2 and unchanging expression of BECN1 caused by TRIM65 siRNA deserve further consideration of a possible intrinsic relationship between autophagy and apoptosis induced by TRIM65 knockdown. It is well documented that Bcl2 interacts with BECN1 and inhibits BECN1-dependent autophagy in mammalian cells (48). Therefore, we speculate that the decrease of anti-apoptotic protein Bcl2 induced by TRIM65 knockdown may upregulate BECN1-dependent autophagy by disrupting the Bcl-2/BECN1 complex.

Our results showed that TRIM65, as an E3 ubiquitin ligase, did not directly degrade autophagy-related proteins in cervical cancer, suggesting that it may target an upstream co-regulator of autophagy and apoptosis. Since p53 expression is linked to regulation of both autophagy and apoptosis (49, 50), p53 protein may play an important role in the potent inhibitory effect of TRIM65 knockdown on cervical cancer by integrating autophagy and apoptosis process. Our investigation further demonstrated that TRIM65 did directly target p53 for ubiquitylation and proteasomal degradation. Importantly, TRIM65 was highly expressed in human cervical cancer tissues and negatively correlated with the expression of p53. This may explain why TRIM65 knockdown regulates autophagy and apoptosis simultaneously to exhibit anti-proliferative activity.

**Figure 6:**
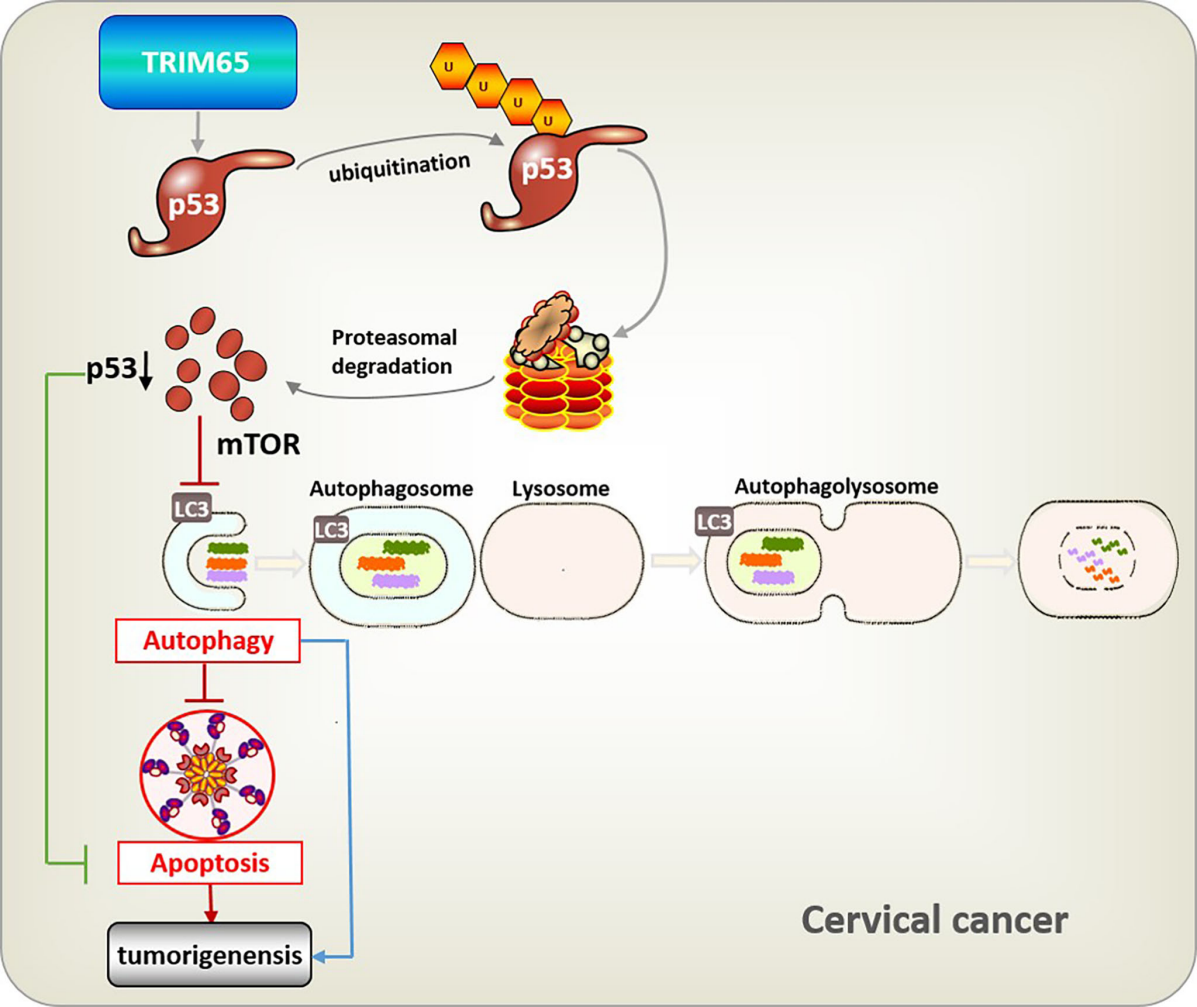
Integrative model illustrating the role of TRIM65 in human cervical cancer. TRIM65-catalized ubiquitylation and degradation of p53, which suppresses directly apoptosis, or causes the inhibition of autophagy through classical mTOR signaling to reduce pathogen degradation, as well as attenuating autophagy-associated apoptosis, thus leading to the tumorigenesis and metastasis of cervical cancer.

In summary, TRIM65-mediated p53 ubiquitination and degradation could directly inhibit apoptosis, and reduce autophagy flux through the classical mTOR signaling pathway which further downregulate autophagy-related apoptosis, and eventually promote carcinogenesis of cervical cancer (**Figure 6**). It is well known that a key role for autophagy in mediating oncovirus infection and tumorigenesis and restoring the autophagic response is of great significance to suppress HPV-mediated diseases (10), especially cervical cancer, thus we think TRIM65 knockdown may at least partially reduce HPV infectivity by promoting autophagy. Taken together, we demonstrated that TRIM65 inhibited autophagic flux and autophagy-related cell apoptosis as an oncogene by targeting p53 degradation in cervical cancer, indicating that TRIM65 knockdown may be useful for preventing HPV infection through promoting autophagy and inhibiting cervical cancer *via* maintaining p53.

## Data Availability Statement

The original contributions presented in the study are included in the article/supplementary material, further inquiries can be directed to the corresponding authors.

## Ethics Statement

The studies involving human participants were reviewed and approved by Ethics Committee of the First Affiliated Hospital of Nanchang University. Written informed consent for participation was not required for this study in accordance with the national legislation and the institutional requirements.

## Author Contributions

X-YW: experiments, data collection and interpretation, and manuscript writing. H-WM: experiments, data collection and interpretation. X-HG, XH: reagents provided and data analysis. Q-MH, Z-PY, JW, H-LT and FZ: data collection and interpretation. H-BX and K-YD: conception and design of the study, data analysis and interpretation, and manuscript revising. All authors read and approved the final version of the manuscript.

## Funding

This work was supported by the National Natural Science Foundation of China (81873659 and 91639106 to H-BX, 81760140 and 81970256 to K-YD, 81800432 to X-HG., and 31960147 to XH), the grant for Jiangxi Provincial Collaborative Innovation Center of Biopharmaceutics and Biotechnology (2015202004 to H-BX) and Jiangxi Provincial Department of Science and Technology, China (20165BCD41001 to H-BX).

## Conflict of Interest

The authors declare that the research was conducted in the absence of any commercial or financial relationships that could be construed as a potential conflict of interest.

## Publisher’s Note

All claims expressed in this article are solely those of the authors and do not necessarily represent those of their affiliated organizations, or those of the publisher, the editors and the reviewers. Any product that may be evaluated in this article, or claim that may be made by its manufacturer, is not guaranteed or endorsed by the publisher.

## Abbreviations

TRIM65, Tripartite motif containing 65; HPV, human papillomavirus; TRIM, Tripartite motif; CQ, Chloroquine.
